# The impact of home-based call on sleep patterns and wellness in genetics and metabolism physicians compared with subspecialists

**DOI:** 10.1016/j.gimo.2024.101819

**Published:** 2024-01-29

**Authors:** Kiley Boone Quintana, Ilana Miller, Debra S. Regier

**Affiliations:** 1 Division of Pediatric Genetics, University of New Mexico, Albuquerque, NM; 2 Genetics and Metabolism, Children’s National Hospital, Washington, DC

**Keywords:** Home-based call, Medical genetics physicians, Pediatric subspecialties, Sleep deprivation, Wellness

## Abstract

**Purpose:**

With increases in precision medicine initiatives and genetically defined rare diseases, the genetics and metabolism workforce is necessary to provide around-the-clock care for patients. Here, we describe the impact that home-based call has on the geneticist and metabolist workforce.

**Methods:**

Physicians from 3 populations were self-identified (pediatric subspecialist, geneticist, metabolist) and completed a survey regarding the impact of home-based call service on their sleep and wellness.

**Results:**

Estimated sleep while serving on home-based call was reduced from 7.5 to 5.4 hours per night. Safety concerns were noted by geneticists and metabolists for themselves (55%) and their families (28%), similar to other subspecialists. Geneticists and metabolists were more likely than other pediatric subspecialists to be worried about their patient’s safety while on home-based call (48% vs 9%). Themes from open-ended questions regarding the impact of home-call included positive responses, decreased access to wellness activities, sleep exhaustion, impact on life responsibility, and impact on mood. Reported coping mechanisms included work-based initiatives, off-loading personal responsibility, and creating personal accommodations.

**Conclusion:**

Institutional-based supports for home-based call were endorsed by only 29% of respondents; thus, interventions at the institutional level would be expected to have a large effect on overall provider wellness.

## Graphical abstract

**Figure:**
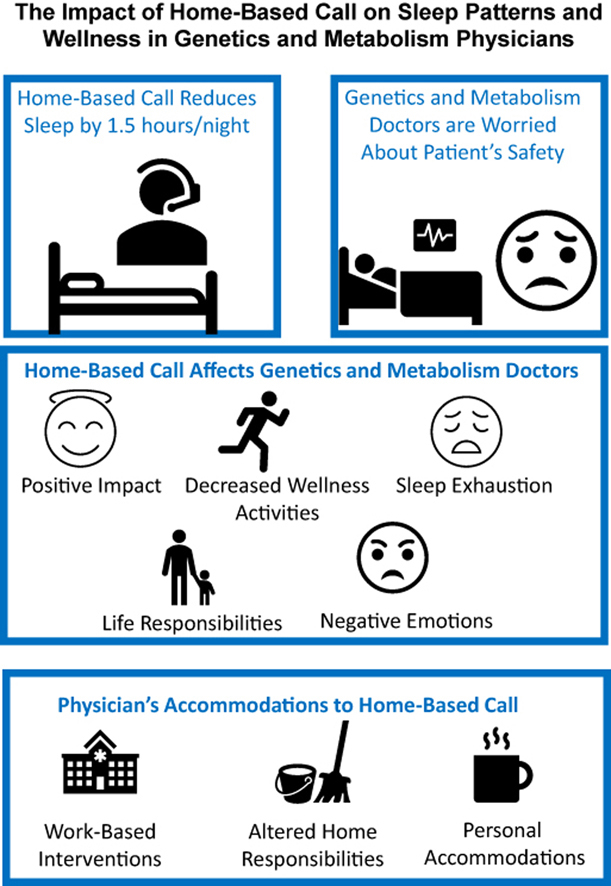

## Introduction

Because the fields of genetics and metabolism have increasing levels of burnout^1^ and increasing demand,^2^ it becomes necessary to assess the work-place components that affect wellness and job satisfaction and help maintain an effective and engaged workforce. These factors could be targeted for support and intervention to improve the overall field of genetics and metabolism.

The effects of sleep deprivation in physicians have been identified and national changes to training have been implemented. Weaver et al^3^ published a summary of studies regarding intern residents’ sleep patterns. Interns limited to 16 working hours per shift experienced reductions in reported significant medical errors, preventable adverse events, and medical errors resulting in a patient death. This relationship between patient safety and fatigue of health care providers contributes to the ongoing discussion on how to support both providers’ and patients’ needs.^3^ Most literature has focused on in-hospital shift time and the effect on sleep deprivation; there is limited research on home-based call to support the in-hospital providers.

The effects of night call on subspecialty populations have also been studied. Chowdhary et al^4^ used Fitbit monitoring for residents over 2 years to determine if night float or home-call had more impact on sleep duration. They concluded that a night float system increased total resident sleep compared with a home-call system.^4^ Ludvigson et al^5^ used a Fitbit to track phone calls and fatigue levels for urology residents for 1 year. They showed an average of 408 minutes of sleep per night for noncall fellows. Fellows without evidence of post-call fatigue had sleep of 368 minutes, whereas fellows with post-call evidence of fatigue had 181 minutes of sleep. On average, each phone call led to 4.71 minutes of reduced sleep.^5^ Ko et al^6^ showed less impact of home-based call for attendings than fellow physicians because of fewer night awakenings. In 2017, Caulley et al^7^ published the experience of otolaryngology residents in a Canadian training program. They tracked home-calls, type of calls, sleepiness scale, and monitored sleep via an actigraph device over a 13-month period. Results showed an average of 7 calls per night with 78.5% for nonurgent issues. Post-call sleep deprivation was associated with the total number of calls after midnight, regardless of location (home vs hospital management).^7^ In 2022, Cho et al^8^ described a study that evaluated 6 nephrology fellows and showed that sleep quality during home-call was restful for “55% of nights.” Thus, not only is sleep duration decreased, it is likely that sleep quality is also reduced.

A 2019 medical genetics survey identified that 73% of geneticists are on-call, with 92% performing telephone and in-person on-call. In-person and telephone call hours averaged 59.1 hours per week, and telephone only coverage averaged 51.3 hours per week.^2^ Thus, we hypothesized that the amount of home-based call could affect the sleep patterns of geneticists and metabolists.

We surveyed physicians who perform home-based call to determine the impact of this work duty on physician sleep patterns. The survey (see Supplementary Information) was distributed to geneticists, metabolists, and pediatric subspecialists. The survey identified an impact on sleep hours per night while on home-based call, identified themes of physician concerns with call responsibilities, and identified coping mechanism used by on-call physicians.

## Materials and Methods

The Children’s National Institutional Review Board (IRB) determined this project is exempt from IRB Review (Study 00499).

We selected a population of subspecialty pediatric physicians that have similarities in the call style and amount of home-based call of geneticists and metabolists. Individuals self-described their on-call specialty. Those who reported medical genetics only are here termed “geneticist.” Those who stated biochemical, metabolic, or genetics and metabolism are termed metabolists. We did not track the location of service, patient population served, or physician diversity. We sent surveys to attending-level physicians; however, we did not prevent fellows or residents from completing the survey. Four individuals noted that they were currently in a training program, but we did not differentiate if this indicated that they were working on home-based call independently or with another more senior physician. Of the 4 in a training program, 1 noted “genetics” as their specialty, and the others were nongenetic or nonmetabolism. For this reason, we did not attempt to separate these 4 individuals from the overall population.

Home-call is when a physician is available while they are outside of the hospital, via texting, phone, pager, or other means of communication. In-house call is defined as being available in the hospital.

### Survey creation

The survey was created in RedCap with support from the Children’s National Children’s Research Institute. Survey questions were reviewed by statistical experts in the Children’s Research Institute (CRI) and 3 physicians before distribution. Survey distribution link and quick response (QR) code were generated by RedCap.

The RedCap survey link and QR code were distributed through the Children’s National Program Director’s and Children’s National Division Chief List Serves with a request to forward to interested physicians. The link was also distributed to all Medical Genetics and Medical Biochemical Genetics (Metabolist) program directors using email addresses available on the Electronic Residence Application Service Site. Recipients were invited to share the link with other interested physicians. Finally, the survey QR code was displayed on a poster at the American Professors for Human and Medical Genetics annual meeting Spring 2023.

The complete RedCap survey is available as an Appendix and will be shared with others for use for future studies regarding this important topic.

### Survey analytics

Survey results were independently analyzed by the 3 authors. Percent responses were calculated and are documented throughout this manuscript.

Short answer responses were evaluated using a qualitative approach. The 3 authors met and read all responses together. After the reading, themes were individually identified and agreed upon as a group until thematic analysis met saturation. Each reader then independently coded the respondents’ remarks. In cases where themes identified did not match, the team of authors met to further discuss the comment. Additional themes were considered after the initial theme lists were created.^9^

## Results

### Demographics

Fifty-one individuals participated in this survey: 29 geneticists and metabolists and 22 other pediatric subspecialists. These specialties included nephrology, cardiac anesthesiology, endocrinology, infectious disease, rheumatology, otolaryngology, and surgery. Two participants did not name their specialty.

### Quantitative outcomes

**Figure 1:**
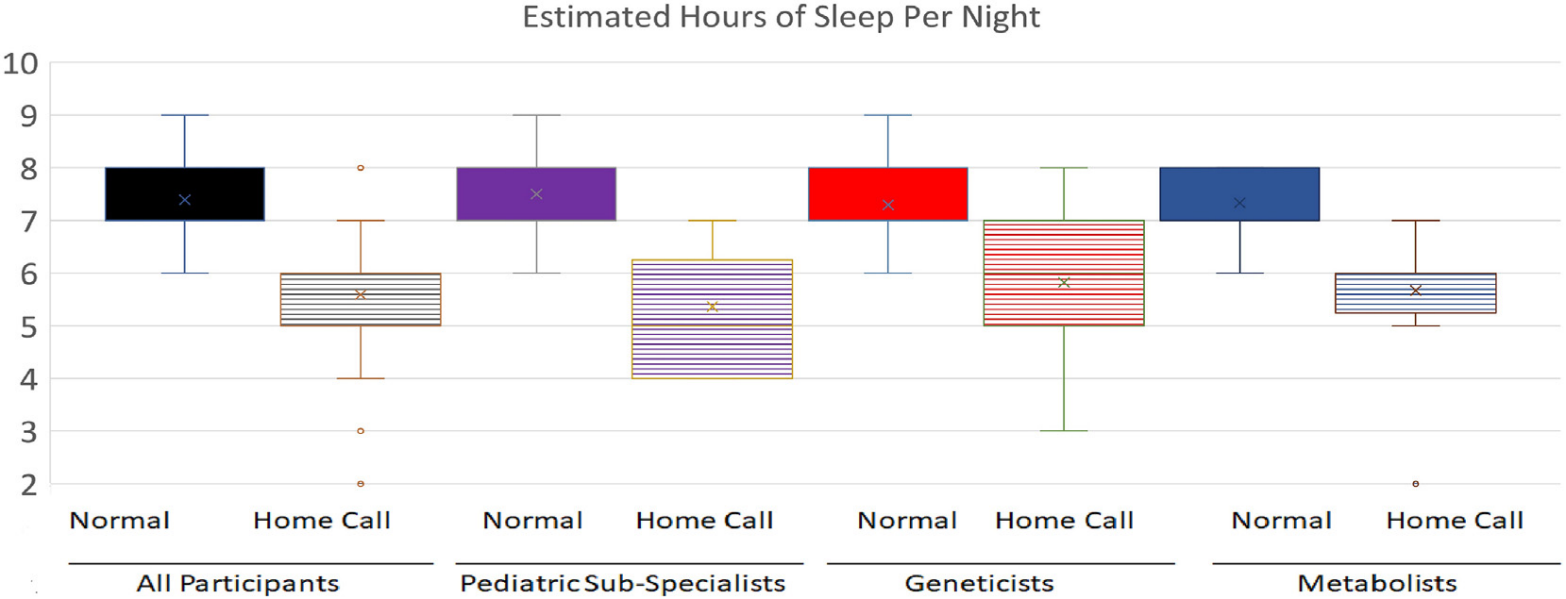
**Sleep differences in home-based call.** Each participant was asked the number of hours per night they sleep on a “normal” (Normal) night and on a “home-based call” night (Home-Call). Responses were averaged (range in answers noted in the figure) with average noted with the “x” in the boxplot. The *y*-axis shows the number of hours of sleep per night. Participants were grouped into all participants (combined pediatric subspecialists, geneticists, and metabolists), pediatric subspecialists (*n* = 22), geneticists (*n* = 17), and metabolists (noted as genetics and metabolism or metabolism on description of call) (*n* = 12).

The average amount of baseline sleep for nongenetic/metabolism subspecialists is 7.5 hours and decreases to 5.4 hours during home-call. For the geneticists, the average sleep at baseline is 7.3 hours and decreases to 5.8 hours during home-call (Figure 1). The sleep effects of geneticists vs metabolists were not significantly different. The average number of nightly conversations between the geneticist/metabolists group and subspecialty group was similar, with 83% of participants in the geneticist/metabolists group having 1 to 5 calls per night, and 86% of the surveyed subspecialists having 1 to 5 nightly conversations.

Geneticists were primarily split between “some effect” and “moderate effect,” both at 31%, when reporting how home-call affects average sleep. Forty-one percent of other subspecialists felt home-call had “some effect” on sleep, and 27% felt home-call had “moderate effect” on sleep. Individuals were surveyed on safety while fatigued after a night of home-call in a variety of realms. Fifty-five percent of both geneticists and other subspecialists were worried about their own safety, such as driving or decision making. Twenty-eight percent of geneticists and 9% of other subspecialists surveyed were worried about their family’s safety.

Forty-eight percent of geneticists compared with 18% of other subspecialists were worried about their patients’ safety after a home-call night.

### Qualitative themes

Five themes were identified when asked “how does home-call affect your personal life, financial well-being, and/or personal well-being?” (Table 1). First, we themed for positive impact, decreased access to wellness, sleep exhaustion, impact on life responsibility, and impact of a negative emotional response (mood, stress, and anxiety). There was a discrepancy between geneticists, metabolists, and other subspecialists regarding positive impact. Twenty percent of metabolists noted a positive impact of call; the positive impact was reported from less than 5% of geneticists and subspecialists. Decreased access to wellness was described in more than 25% of all responses; sleep exhaustion was noted in 25%.

**Table 1:** How does home-call affect your personal life, financial well-being, and/or personal well-being?

Theme	Respondent Comments
Positive impact	Also allows me to try to get exercise in during call times.
	Call is one of my favorite parts of my job. It’s consistently interesting and rewarding.
	Much better now that I...can refer them to Biochem and go back to sleep.
	Like it better than in-hospital call in that [I] see spouse.
Decreased wellness activities	It makes it difficult to enjoy free time and do rejuvenating activities because I am too tired, so I resort to things that aren’t healthy in the long run.
	I am not able to exercise as much as I would on a non-Home-call week.
	I find it mainly increases my stress/anxiety level whenever I am on-call, as it is harder to relax.
	I often get sick.
Sleep exhaustion	Partner also suffered from sleep interruptions due to pager wake ups and phone calls in middle of the night.
	It still affects my sleep and mood.
	It is quite disruptive. Frequent calls in the middle of the night, even short ones, lead to several hours of lost sleep overnight and also interfere with the sleep quality of other household members.
	The fatigue can be significant and hard to recover from.
Life responsibilities	Huge impact on childcare and family dynamics in terms of time spent working.
	Home-call weeks are expensive.
	It takes me away from participating or being really present in my children’s important events. This all leads to a lot of guilt.
Negative emotional responses	My mood is less positive when I am sleep deprived.
	It causes stress because I feel I am on a leash... So, I always feel I need to check my pager, it is not good for my mental health.
	Children do not care for my weeks of call and said they would never be doctors.
	Being on-call gives me a lot of anxiety... I need a full weekend of doing nothing to rest and recharge after a week of home-call.

Respondents were asked to comment on how home-based call affects their lives. Responses were evaluated for themes (positive impact, decreased wellness activities, sleep exhaustion, life responsibilities, and negative emotional responses). Example are shown in each theme.

Second, we asked “please share some of your coping mechanisms for how you’ve learned to minimize the distraction of home-call or improve your ability to manage this responsibility” (Table 2). Answer themes included work-based interventions, off-loading personal responsibility, and creating personal accommodations, most commonly personal accommodations to cope. This included using take-out for food, rescheduling exercise, and increasing caffeine or food consumption. Interestingly, only 17 of 58 responses noted how work-based interventions supported their coping mechanisms. These included having call-free time periods, patient load changes, and coverage by others.

**Table 2:** Respondent comments on coping mechanisms^a^

Theme	Respondent Comments
Work-based interventions	There are no personal coping mechanisms that have helped me with this. Systemic changes have helped—like reducing the number of on-call days and having another person respond to the call first.
	We are now mostly on-call with a fellow which has dramatically changed the quality of home-call.
	Our division tries to put attendings on night call on a day they don’t have clinic the next morning.
	Shorter notes.
Decreased or altered home responsibilities	Try to get home responsibilities done before call to minimize need to cook dinner, do laundry, etc.
	I make sure my family knows that I could be called at any time and that I cannot just ignore a call.
	Minimizing expectations of non-call activities (eg, decreased meal cooking, defer laundry).
	Attempt to decrease responsibilities following call.
Personal accommodations	Planning to limit academic deadlines/requirements during the week.
	Usually skip self-care (exercise/time with spouse).
	I don’t schedule any other activities for the week that I am on-call
	I neglect my personal life and self-care. I also drink a lot of caffeine and complain to my colleagues.

Respondents were asked to comment on interventions that they found supportive in performing home-based call. Responses were evaluated for themes (work-based interventions, decreased or altered home responsibilities, and personal accommodations). Example are shown in each theme.

a Please share some of your coping mechanisms for how you’ve learned to minimize the distraction of home-call or improve your ability to manage this responsibility.

## Discussion

With the burnout levels reported by Arnold et al^1^ in the genetics and metabolism communities, the current workforce demand for more trained geneticists and metabolists,^2^ and the impact of sleep on burnout,^10^ we explored the impact of sleep on the genetics and metabolism physician communities and what best practices could improve outcomes. Here, we show a reduction in the total sleep latency on home-based call and fatigue after home-based call. With respondents on-call from every other night to every 10th night, the range of home-based call was broad, as expected by the field. Those on-call more frequently had fewer nightly phone calls than those on-call less often. The effect of this sleep loss and burnout risks has been well described.^10^

The data showed that geneticists and metabolists had similar experiences with reported total sleep duration while on home-based call. Those on metabolism call reported more stress because of the patient acuity, time-sensitive nature, and impact on life. However, the positive comments about call being interesting and leading to job satisfaction was shared more by the metabolists. The overall impact of call on sleep was similar between all 3 groups of physicians.

Eighteen percent of subspecialists stated they were concerned for patient safety during home-call. In comparison, 50% of metabolists and 47% of geneticists were concerned for patient safety while on home-based call. The reason for this increased concern from other home-call providers was not fully explored in this research. Literature exists that considers the resources needed for rare disease patient safety in the health care system and the need for additional resources to support in-hospital providers who are not rare disease experts.^11^ Future research is essential to better understand these concerns. There is also a need for tool and resource development to support geneticists and metabolists so that in-hospital providers can be better prepared to care for these patients.

The qualitative themes identified organizational influences on the home-based call stressors in 29% of responses. Thus, this area is a prime area of advocacy across geneticists, metabolists, and subspecialist practice groups. Implementation mechanisms that were viewed positively included having a triage mechanism, the use of trainee level providers as first call, rest or decreased workload after home-based call, and changing medical record requirements. As with any intervention, balancing measures need to be identified. For example, with a triage system, the provider concern for patients may remain elevated, and by having fellows as first line call, their wellness and retention in the field may be affected. Finally, documentation that is not fully aligned with institutional needs may lead to reduced reimbursement and decreased profitability of these field.

Based on this work, it is essential that divisions across specialties consider how to best support home-based call to decrease burnout of providers and ensure patient safety. Interestingly, the number of metabolists with positive comments about call exceeded that of geneticists and subspecialists. Thus, there is some job satisfaction that comes from home-based call. One respondent stated, “call is one of my favorite parts of my job. It’s consistently interesting and rewarding.” Based on this finding, it is important to retain the positive job satisfaction while decreasing the risk of home-call on burnout and workforce retention.

## Data Availability

The survey templates will be shared with any individuals who would like to pursue additional evaluation of home-based impacts of sleep.

## Conflict of Interest

The authors declare no conflicts of interest.
