# Supplementary material for: The impact of home-based call on sleep patterns and wellness in genetics and metabolism physicians compared with subspecialists
Source: Genet Med Open. 2024 Jan 29;2:101819. doi: 10.1016/j.gimo.2024.101819 (PMC11613610; doi:10.1016/j.gimo.2024.101819)
Supplement: SleepSurveyQuestions.pdf [file mmc1.pdf]

Form 1

Please complete the survey below.

Thank you!

1) How long ago did you complete your first residency program?

- ◯ I have not yet completed my first residency
- ◯ I am currently in a fellowship or second residency
- ◯ 0-2 years
- ◯ 3-5 years
- ◯ 6-15 years
- ◯ 16-25 years
- ◯ 26-35 years
- ◯ More than 36 years

2) In an average night when NOT on-call (home or in-hospital), how many hours of sleep would you have?

- ◯ Less than 4 hours per night
- ◯ 4
- ◯ 5
- ◯ 6
- ◯ 7
- ◯ 8
- ◯ 9
- ◯ 10
- ◯ More than 10 hours per night

3) In an average YEAR, how frequently s are you on-call IN PERSON (i.e. at the hospital or clinic)

- ◯ 0 (never)
- ◯ Monthly (equivalent to 1.5 weeks per year)
- ◯ Weekly (equivalent to 7 weeks per year)
- ◯ Twice per week (equivalent to 14 weeks per year or about Q4 call)
- ◯ More than twice per week (equivalent to Q3 or Q2 call)

4) During an IN-HOUSE call night, how many hours of sleep per night would you AVERAGE over the course of a year?

- ◯ 0 (no sleep)
- ◯ 1 hour
- ◯ 2 hours
- ◯ 3 hours
- ◯ 4 hours
- ◯ 5 hours
- ◯ 6 hours
- ◯ 7 hours
- ◯ 8 hours
- ◯ 9 hours
- ◯ 10 hours
- ◯ More than 10 hours

5) In an average YEAR how frequently are you on HOME CALL (i.e. taking calls away from the hospital)

- ◯ 0 (never)
- ◯ Monthly (equivalent to 1.5 weeks per year)
- ◯ Weekly (equivalent to 7 weeks per year)
- ◯ Twice per week (equivalent to 14 weeks per year or about Q4 call)
- ◯ Four times per week (50% on-call nights)
- ◯ Most nights (about 100% on-call nights)

6) During a Home Call night, how many hours of sleep per night do you AVERAGE over the course of a Year?

- ◯ 0 (no sleep)
- ◯ 1 hour
- ◯ 2 hours
- ◯ 3 hours
- ◯ 4 hours
- ◯ 5 hours
- ◯ 6 hours
- ◯ 7 hours
- ◯ 8 hours
- ◯ 9 hours
- ◯ 10 hours
- ◯ more than 10 hours
- ◯ Not Applicable, I do not do Home call.

7) In an AVERAGE Night of Home Call (5pm to 8am), how many calls in total do you receive?

- ◯ 0
- ◯ 1-5
- ◯ 6-10
- ◯ 11-20
- ◯ More than 20 calls per night
- ◯ Not applicable, I do not take home call

8) In an AVERAGE night of home call we realize there is a variety of call lengths. Please choose the THREE answers that describe the a) shortest call, b) average call, and c) longest call time (in minutes)

- ☐ 1 minute ☐ 2-4 minutes
- ☐ 5-7 minutes ☐ 8-14 minutes
- ☐ 15-20 minutes ☐ 21-30 minutes
- ☐ 31-60 minutes ☐ more than 61 minutes

9) What is the LONGEST duration of time are you available in-person and by home call (sequential days)?

- ◯ 12 hours
- ◯ 1 day
- ◯ 2-5 days
- ◯ 6-8 days
- ◯ 9-14 days
- ◯ more than 14 days sequentially
- ◯ Not applicable, I do not take home call

10) How much do you feel that home call affects your sleep on an AVERAGE Home call night?

- ◯ None
- ◯ Little effect
- ◯ Some effect
- ◯ Moderate effect
- ◯ Extensive effect

11) Following a HOME call night, have you ever been worried for the safety of any of the following because of your fatigue? (Select all that apply)

- ☐ Yourself (i.e. driving, decision making, etc)
- ☐ Your patients
- ☐ Your family
- ☐ Your community within the hospital setting
- ☐ Your community outside of the hospital setting
- ☐ Other (please list in the text box supplied in the next questions)
- ☐ I have never felt unsafe following a HOME call night

12) What are some of the coping mechanisms you employ following home call nights? (Select all that apply)

- ☐ Decreased patient care responsibilities
- ☐ Later start time
- ☐ Early stop time
- ☐ Decreased personal time/self care time
- ☐ Increased caffeine or food consumption
- ☐ Increased napping/sleeping
- ☐ No coping mechanism used
- ☐ Other (please list in next question)

13) Please share some of your coping mechanisms for how you've learned to minimize the distraction of home call or improve your ability to manage this responsibility.

____

14) How does Home Call affect your personal life, financial well being, and/or personal well being?

____

15) How do you identify? (choose all that apply)

- ☐ Woman
- ☐ Man
- ☐ Transgender/Trans woman
- ☐ Transgender/Trans man
- ☐ Non-Binary
- ☐ None of the above describe how I identify
- ☐ Prefer not to reply

16) Which of the following describe you? (choose all that apply)

- ☐ American Indican or Alaks Native
- ☐ Asian
- ☐ Black or African American
- ☐ Native Hawaiian or Pacific islander
- ☐ White
- ☐ Hispanic
- ☐ None of these describe me.

17) What is the medical specialty for which you perform HOME call?

____
